# Conductive Atomic Force Microscopy of Semiconducting Transition Metal Dichalcogenides and Heterostructures

**DOI:** 10.3390/nano10040803

**Published:** 2020-04-22

**Authors:** Filippo Giannazzo, Emanuela Schilirò, Giuseppe Greco, Fabrizio Roccaforte

**Affiliations:** CNR-IMM, Strada VIII, 5-5121 Catania, Italy; emanuela.schiliro@imm.cnr.it (E.S.); giuseppe.greco@imm.cnr.it (G.G.); fabrizio.roccaforte@imm.cnr.it (F.R.)

**Keywords:** conductive atomic force microscopy, transition metal dichalcogenides, Schottky barrier, conductivity, heterostructures

## Abstract

Semiconducting transition metal dichalcogenides (TMDs) are promising materials for future electronic and optoelectronic applications. However, their electronic properties are strongly affected by peculiar nanoscale defects/inhomogeneities (point or complex defects, thickness fluctuations, grain boundaries, etc.), which are intrinsic of these materials or introduced during device fabrication processes. This paper reviews recent applications of conductive atomic force microscopy (C-AFM) to the investigation of nanoscale transport properties in TMDs, discussing the implications of the local phenomena in the overall behavior of TMD-based devices. Nanoscale resolution current spectroscopy and mapping by C-AFM provided information on the Schottky barrier uniformity and shed light on the mechanisms responsible for the Fermi level pinning commonly observed at metal/TMD interfaces. Methods for nanoscale tailoring of the Schottky barrier in MoS_2_ for the realization of ambipolar transistors are also illustrated. Experiments on local conductivity mapping in monolayer MoS_2_ grown by chemical vapor deposition (CVD) on SiO_2_ substrates are discussed, providing a direct evidence of the resistance associated to the grain boundaries (GBs) between MoS_2_ domains. Finally, C-AFM provided an insight into the current transport phenomena in TMD-based heterostructures, including lateral heterojunctions observed within Mo_x_W_1–x_Se_2_ alloys, and vertical heterostructures made by van der Waals stacking of different TMDs (e.g., MoS_2_/WSe_2_) or by CVD growth of TMDs on bulk semiconductors.

## 1. Introduction

In the last years, transition metal dichalcogenides (TMDs) have attracted an increasing scientific interest because of their unique and tunable electronic structure, holding great promise for next generation applications in electronics and optoelectronics [1,2]. These layered materials are composed by the stacking of MX_2_ trilayers (M = Mo, W, etc. and X = S, Se, Te, etc.) held together by van der Waals bonds. Each trilayer consists of a layer of hexagonal-packed transition-metal atoms (M) sandwiched between two layers of chalcogen atoms (X), with a strong intralayer covalent bond between the different atoms. Depending on the composition and internal structure of the individual trilayers, the electronic behavior of TMDs can range from insulating to semiconducting and semi-metallic. Furthermore, the optical and electronic properties of these layered materials depend on the stacking order and number of layers [3]. For instance, bulk or multilayer MoS_2_ exhibit an indirect band gap of ~1.3 eV, whereas monolayer MoS_2_ presents a direct band gap of 1.8–1.9 eV. A similar behavior also occurs in other semiconducting TMDs. The tunable band gap along with the high quantum efficiency and optical absorption make TMDs interesting for optoelectronics [1]. In addition, due to the superior electrostatic control of channel conductance in field effect transistors (FETs) with an ultra-thin TMD body [4,5], these 2D semiconductors are currently regarded as strategic materials for the post-Si complementary metal oxide semiconductor (CMOS) technology [6]. Very interesting performances in terms of the on/off current ratio (10^6^–10^8^) and a subthreshold swing approaching the ideal value (60 meV/decade) have been already demonstrated in the early studies on single layer [4] and multilayer MoS_2_ FETs [5]. More recently, ultra-scaled MoS_2_ FETs with a 1 nm gate length [7] or with a sub −10 nm channel length [8] have been reported, further supporting the expectations for MoS_2_ transistors to further extend the validity of Moore’s law [9]. In addition, as replacements of silicon for the CMOS technology, TMDs are also currently employed as building blocks of new transistor concepts based on vertical van der Waals heterostructures [10,11,12], or in-plane heterojunctions obtained by engineering the TMDs composition [13] or even the dielectric environment of the 2D semiconductors [14].

To date, many of these TMD-based (opto)electronic devices have been fabricated using small flakes obtained by mechanical exfoliation from the parent bulk crystals, due to the ease of preparation in academic laboratories [4,10]. However, the lateral size (few micrometres) and the low density of flakes produced by this approach are unsuitable for practical applications, and large area deposition methods of TMDs are required for a scale-up of these technologies. In the last years, many progresses have been made in the growth of TMDs by chemical vapor deposition (CVD). The direct CVD growth of TMDs, in particular MoS_2_, on noncatalytic insulating or semiconducting substrates (including SiO_2_ [15,16,17], sapphire [18], and GaN [19]) is possible at relatively low temperatures in the range from 700 to 900 °C. This represents an important aspect for the integration of this process in the CMOS devices fabrication flow. The CVD growth of highly uniform monolayer TMDs on 100 mm [20] and, more recently, 150 mm wafers [21] have been demonstrated. Furthermore, alternative chemical deposition methods, e.g., atomic layer deposition (ALD) [22], or physical depositions, e.g., molecular beam epitaxy (MBE) [23] and pulsed laser deposition (PLD) [24], are currently explored to achieve layer-by-layer growth of MoS_2_ on a large area, up to the wafer scale.

Table 1 summarizes some of the most relevant achievements in the TMD devices and materials growth in the last years.

In spite of these recent progresses, several issues need to be addressed to fully exploit the potential of TMDs in real applications. In addition to the request of optimized thickness uniformity, defectivity, and doping of the deposited films, a further challenge for materials growth is represented by the engineering of TMD vertical or lateral heterojunctions [12]. On the other hand, low resistance ohmic contacts and selective-area doping of TMDs are two major requirements for the fabrication of devices based on these materials.

**Figure 1:**
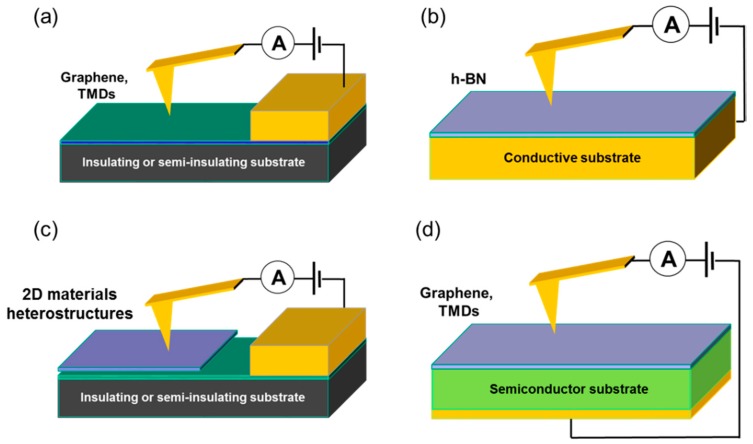
Schematic illustration of conductive atomic force microscopy (C-AFM) applications in two dimensional (2D) materials characterizations. (**a**) Lateral current transport in semi-metallic graphene or semiconducting TMDs. (**b**) Tunneling current through insulating hexagonal boron nitride (h-BN). (**c**) Current transport in 2D materials vertical (or lateral) heterostructures. (**d**) Current injection in the heterojunctions of 2D materials (graphene or TMDs) with bulk semiconductors.

High resolution structural, chemical, and electrical characterizations revealed that the overall electronic properties of TMD_S_ are ultimately determined by peculiar nanoscale defects/inhomogeneities (e.g., point or complex defects, thickness fluctuations, grain boundaries, wrinkles, etc.), which can be intrinsic of these materials or dependent on the growth method, on the substrates and on the device fabrication processes. In this context, electrical atomic force microscopy methods, such as conductive atomic force microscopy (C-AFM), scanning capacitance microscopy (SCM), Kelvin probe force microscopy (KPFM), and scanning microwave impedance microscopy (SMIM) proved to be essential tools to understand the nature and electrical activity of defects/nonuniformities in 2D materials, and more specifically in TMDs [34,35,36,37,38,39]. In particular, C-AFM allows performing a high-resolution current mapping and local current-voltage (I–V) characterization at the nanoscale [40,41,42,43]. Hence, it is the method of choice to investigate the mechanisms of current injection from contacts to 2D materials [44,45], the lateral homogeneity of conductivity of 2D semimetals (graphene) [46,47,48], and semiconductors (TMDs) [44], as well as the transversal current transport across thin dielectric films [49,50] or 2D insulators (such as h-BN) [51,52,53]. Furthermore, C-AFM allows investigating the vertical current injection across the van der Waals heterostructures of 2D materials [12] or their heterojunctions with bulk semiconductors [19,54,55,56]. Figure 1 schematically illustrates these possible applications of C-AFM in the characterization of 2D materials and heterostructures.

This paper reviews some relevant applications of the C-AFM technique to the investigation of nanoscale transport properties in TMDs, discussing the implications of the local phenomena in the overall behavior of devices fabricated on these materials.

Section 2 includes recent studies of the current injection mechanisms at the metal/TMDs junctions. Nanoscale resolution current spectroscopy and mapping by C-AFM provided information on the Schottky barrier height (SBH) uniformity and shed light on the mechanisms responsible for the Fermi level pinning commonly observed at the metal/TMDs interface. The application of this approach to nanoscale mapping of the SBH distribution in MoS_2_ thin films subjected to oxygen plasma prefunctionalization is illustrated, and the practical implications of these results in the realization of ambipolar MoS_2_ transistors are also demonstrated.

Section 3 presents experiments on local conductivity mapping in monolayer MoS_2_ grown by CVD, providing a direct evidence of the role of grain boundaries (GBs) between MoS_2_ domains on the overall current transport in these layers.

Finally, Section 4 presents C-AFM studies of the current transport in TMD-based heterostructures, including lateral heterojunctions present in TMDs alloys, and vertical van der Waals heterojunctions formed by the stacking of different TMDs and of TMDs with bulk semiconductors.

## 2. Schottky Barrier Height Mapping at Metal/TMDs Junction

The lack of out-of-plane bonds in TMDs initially led to the expectation that the Schottky barrier at the interface between metal contacts and TMDs would approach the Schottky–Mott limit of weak pinning [57]. However, contrary to this expectation, experiments showed the opposite behavior, i.e., the SBH for most semiconducting TMDs is only weakly dependent on the metal work function, and this effect has been attributed to a Fermi level pinning [58,59,60,61]. Clearly, this phenomenon has strong implications on TMDs-based transistors, as the current injection/extraction from the source/drain contacts rules the overall device behavior [62]. As an example, in the case of MoS_2_ thin films (produced either by exfoliation or by CVD) most of the elementary metals exhibit a Fermi level pinning close to the MoS_2_ conduction band, resulting in SBH values for electrons injection ranging from 20–60 meV for low work function metals (Sc, Ti, etc.) to 150–250 meV for high work function ones (Ni, Pt, etc.) [58]. As a matter of fact, the low SBH for electrons translates into a high SBH for holes. Hence, n-type FETs with an electron accumulation channel can be easily obtained with unintentionally n-type doped MoS_2_, whereas the fabrication of MoS_2_ FETs with the complementary p-type behavior is challenging, due to the difficulty to inject holes in the inversion channel [63,64]. Contrary to the case of MoS_2_, a p-type behavior is commonly observed in monolayer WSe_2_ FETs [26], whereas ambipolar transistors have been demonstrated using a few layers of MoTe_2_ [65]. In spite of these differences, the electrical behavior of TMDs-based FETs is generally dominated by the Schottky barrier of source and drain contacts.

The origin of the Fermi level pinning at the junction between metals and TMDs is currently an object of debate. According to the commonly accepted models of Fermi level pinning at metal/semiconductor interfaces, this effect can be ascribed either to metal-induced gap states (MIGS) [66] or to the disorder induced gap states (DIGS) [67]. In the DIGS model, the Fermi level pinning is due to states in the electronic structure of the host semiconductor associated to defects in its crystalline structure. On the other hand, the MIGS model predicts that the states in the gap are induced by the interaction with the metal, i.e., the metal dominates over the substrate in pinning the Fermi level. Recently, ab-initio simulations of defect-free TMDs showed that the metal/TMD junctions largely follow the MIGS model, similar to three-dimensional semiconductors despite their different structure and bonding [59]. In this context, Fermi level depinning by the insertion of an ultra-thin tunnel barrier (such as monolayer h-BN) between contacts and MoS_2_ has been recently reported [30]. The main function of this barrier layer is increasing the physical separation between the MoS_2_ and the contact electrode, thereby minimizing the metal/MoS_2_ interfacial interaction responsible for the creation of MIGS. On the other hand, early nanoscale electrical investigations (based on scanning tunneling microscopy) highlighted the possible role of the defects present at the MoS_2_ surface in the Fermi level pinning phenomenon [68].

As a matter of fact, SBH mapping with nanoscale resolution is required to disentangle the effect of surface (or near surface) defects in the TMD materials from that of metal-induced gap states. To this purpose, the conductive tip of C-AFM has been employed as a nanoscopic metal electrode to record local I-V characteristics on the TMDs surface, from which the SBH was quantitatively evaluated [44,45,69]. Contacting the TMD surface with a sliding metal tip presents the additional advantage of excluding eventual reactions that have been reported at the metal/TMD interface (for some metal species) when the contact is fabricated by evaporation or sputtering [70,71]. In this way, the effect of the interaction with a metal electrode in close proximity to the TMD surface can be investigated. In the following subsection, recent studies of the Fermi level pinning in TMDs using C-AFM are discussed.

### 2.1. C-AFM Investigations of Fermi Level Pinning in TMDs

**Figure 2:**
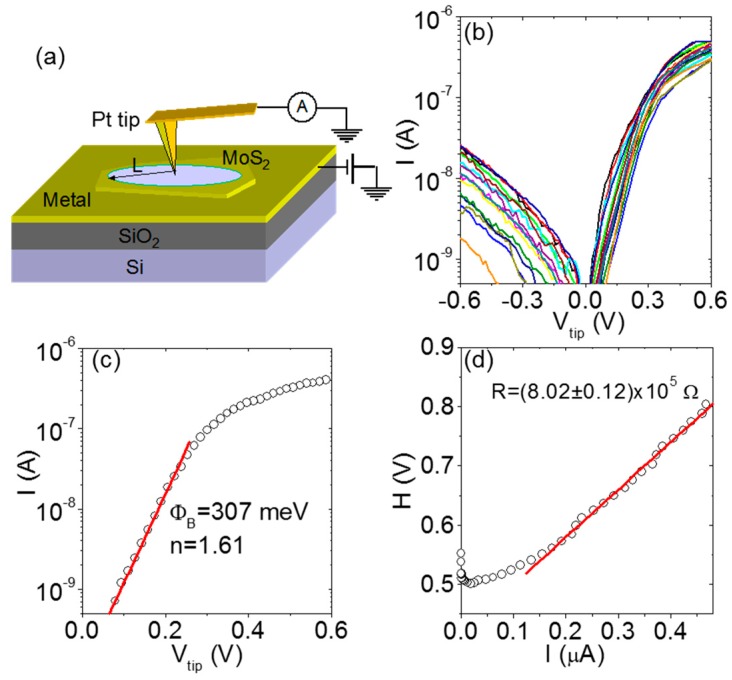
(**a**) Schematic of the experimental setup for C-AFM measurements on MoS_2_. (**b**) Set of 25 *I*-*V_tip_* characteristics measured on a 500 × 500 nm array of tip positions with ~100 nm spacing on MoS_2_. (**c**) Representative forward bias *I*-*V_tip_* characteristic from this set of measurements and fit with the thermionic emission law to extract the Schottky barrier height (SBH) and ideality factor. (**d**) The *H* function plot for the determination of the series resistance *R*. Figures adapted with permission from Reference [44], copyright from the American Physical Society 2015.

The C-AFM technique was firstly employed in the current-voltage (I-V) spectroscopy mode to investigate the current injection to the surface of multilayer MoS_2_ exfoliated on a SiO_2_ substrate [44]. Local I-V measurements were performed under ambient conditions using a Pt coated Si tip (curvature radius *r*_tip_ ≈ 10 nm) connected to a high sensitivity current amplifier, while a bias was applied to a macroscopic front contact, as schematically illustrated in Figure 2a. Figure 2b shows a set of 25 *I*-*V_tip_* curves (with *V_tip_* as the bias referred to the tip) measured on the MoS_2_ surface on a 0.5 × 0.5 μm area. All the curves are asymmetric from a positive (forward) to negative (reverse) bias, indicating a Schottky behavior of the Pt/MoS_2_ contact. A representative forward *I*-*V_tip_* characteristic from this array of measurements is reported in Figure 2c. In the semilog-plot, a linear increase of the current over more than two decades (from 1 × 10^−10^ to 5 × 10^−8^ A) is observed, followed by a saturation. The SBH (Φ*_B_*) and the ideality factor (*n*) for the nanoscopic Pt/MoS_2_ contact were evaluated by fitting the low voltage region of the forward *I*-*V_tip_* curve with the thermionic emission law:

(1) $$I=AA^{*}T^{2}\exp\left(-\frac{q\Phi_{B}}{k_{B}T}\right)\exp\left(\frac{qV_{tip}}{nk_{B}T}\right)$$

where *q* is the electron charge, *k_B_* is the Boltzmann constant, *T* is the absolute temperature (*T* = 300 K), *A* = *πr*_tip_^2^ is the tip contact area, and A* is the Richardson constant of multilayer MoS_2_ [44]. In particular, Φ*_B_* = 307 meV and *n* = 1.61 were determined from the intercept and the slope of the linear fit of the ln(*I*)-*V_tip_* characteristic for *V_tip_* < 0.3 V. The current saturation observed on the semilog-scale at higher bias values is due to a series resistance contribution *R*.

**Figure 3:**
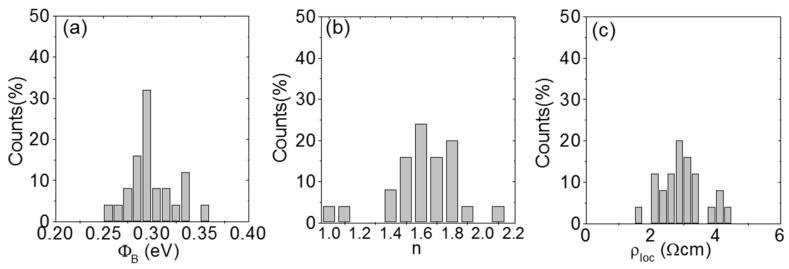
Histograms of the local Schottky barrier height Φ*_B_* (**a**), ideality factor *n* (**b**), and resistivity *ρ*_loc_ (**c**) extracted from the array of *I*-*V_tip_* characteristics in Figure 2b. Figures adapted with permission from Reference [44], copyright from the American Physical Society 2015.

Since the downward curvature in the high voltage region of the *I*-*V_tip_* curves depends both on *n* and *R*, the Cheung’s method [72] was applied to evaluate the *R* contribution. In this method, the function *H* is defined as *H* = *V_tip_*_−_*nk_B_T/q* ln[*I*/(AA*T^2^)], which depends on *I* as *H* = *n*Φ*_B_* + *IR*. Figure 2d shows a plot of *H* vs. *I* obtained from the forward bias *I*-*V_tip_* characteristic in Figure 2c. *R* = (8.02 ± 0.12) × 10^5^ Ω was evaluated by the slope of the linear fit for current values larger than 0.1 μA. The R value is mainly due to the spreading resistance *R*_spr_, associated to current spreading into multilayer MoS_2_ from the nanoscale contact. *R*_spr_ is related to the local resistivity *ρ*_loc_ of MoS_2_ under the tip as *R*_spr_ = *ρ*_loc_/(4r_tip_), being r_tip_ the contact radius. Hence, the local resistivity *ρ*_loc_ could be estimated from the *R* values measured at each tip position. By performing the same analysis on the full set of *I*-*V*_tip_ characteristics of Figure 2b, the distributions of the local SBHs, ideality factors, and resistivity values at the different tip positions on MoS_2_ were determined. The histograms of the Φ*_B_*, *n*, and *ρ*_loc_ values are reported in Figure 3a–c, respectively.

The distribution in Figure 3a shows an average SBH of 300 meV with a standard deviation of 24 meV. This average value is in good agreement with the SBH evaluated for deposited Pt contacts on multilayer MoS_2_ [58]. According to the Schottky–Mott theory, the ideal SBH value for a metal/semiconductor contact without Fermi level pinning is expressed as:

Φ*_B_* = *W*_M_ − χ (2)

where *W*_M_ is the metal work function and χ is the semiconductor electron affinity. In the specific case of Pt and multilayer MoS_2_, Φ_Μ_ ≈ 5.4 eV and χ ≈ 4.1 eV, result in an ideal Φ*_B_* ≈ 1.3 eV. Clearly, the experimental SBH values are much lower than the ideal one. The histogram in Figure 3b shows that *n* is close to unity only on ~10% of the investigated MoS_2_ area, whereas the average value of *n* is 1.60 with a standard deviation of 0.23. Generally, the deviation of *n* from unity indicates that the current transport is not perfectly described by the thermionic emission theory and can be ascribed to the presence of surface states [73]. Finally, an average resistivity of 2.99 Ωcm with a standard deviation of 0.68 Ωcm was estimated from the distribution in Figure 3c. The lateral variations of *ρ*_loc_ can be ascribed to inhomogeneities in the carrier concentration and/or in the carrier mobility of MoS_2_.

Since these C-AFM analyses were carried out under ambient conditions, the measured SBH distribution can be affected by the presence of a water meniscus under the tip, which is known to cause a degradation of the lateral resolution. In this context, performing C-AFM under a high vacuum or within an environmental chamber has been shown to allow current mapping and spectroscopy with a greatly improved resolution [74].

**Figure 4:**
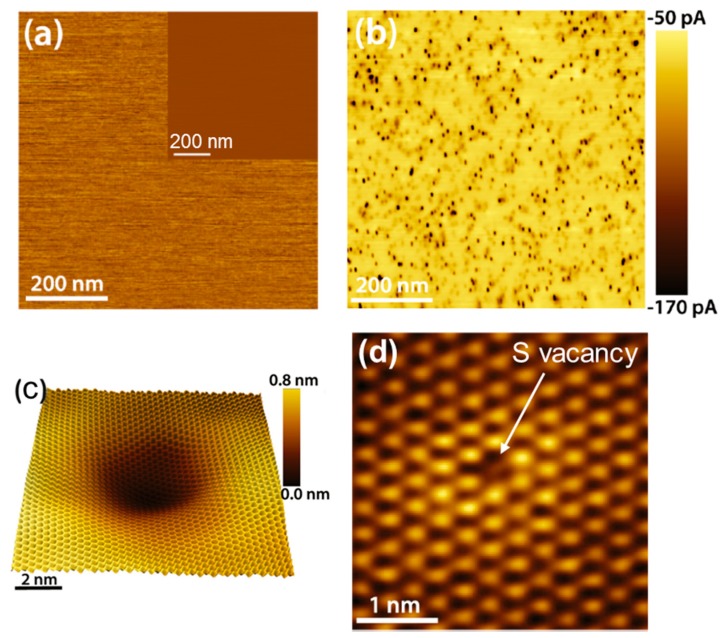
(**a**) Lateral force microscopy (LFM) and topography (insert) image of the MoS_2_ surface. (**b**) Simultaneously recorded C-AFM image. (**c**) Scanning tunneling microscopy (STM) image of a subsurface defect, corresponding to one of the high current spots in the panel (**b**). (**d**) STM image of a sulfur vacancy in the outermost sulfur layer. Images adapted with permission from Reference [45], copyright from the American Chemical Society 2017.

Recently, high resolution current mapping by an environmental chamber C-AFM, combined with atomic resolution scanning tunneling microscopy (STM), provided further insight on the nature of defects responsible for the Fermi level pinning in MoS_2_ [45] and other TMDs (MoSe_2_, MoTe_2_, WS_2_, and WSe_2_) [75]. Figure 4a shows a lateral force microscopy (LFM) and the corresponding AFM topographic image (inset) of a freshly cleaved MoS_2_ sample, showing a smooth surface in both images [45]. On the other hand, the simultaneously recorded C-AFM map (Figure 4b) shows a laterally inhomogeneous current injection through the nanoscopic contact between the conductive tip (made of boron doped diamond) and the MoS_2_ surface. Multiple dark circular features (with radii ranging between 3 and 4 nm) are present in this image, with a higher current than the surrounding areas, indicating a lower tip/MoS_2_ contact resistance. The surface density of these features was found in the range between 10^10^ and 10^11^ cm^–2^, depending on the sample and location. Atomic resolution scanning tunneling microscopy (STM) analyses of the MoS_2_ surface were also carried out to understand the nature of the high current spots in the C-AFM images. The STM maps revealed the presence of characteristic features appearing as circular depressions on which the MoS_2_ lattice is superimposed (see Figure 4c). The typical lateral size of these depressions was up to 5 nm and their areal density was (8 ± 3) × 10^10^ cm^−2^, similar to the density of conductive features in the C-AFM images. The absence of such depressions in the topographic and LFM images (Figure 4a) suggested that the observed features in the STM maps are electronic in nature and induced by subsurface defects. They were associated to Mo-vacancies or Mo-substitutional defects, located below the outermost S layer [45]. In addition to these subsurface defects, sulfur vacancies (see Figure 4d) were found to be ubiquitously present in the topmost S layer of MoS_2_, with an areal density of (7 ± 4) × 10^12^ cm^−2^. No specific features corresponding to these point defects were observed in the C-AFM map.

**Figure 5:**
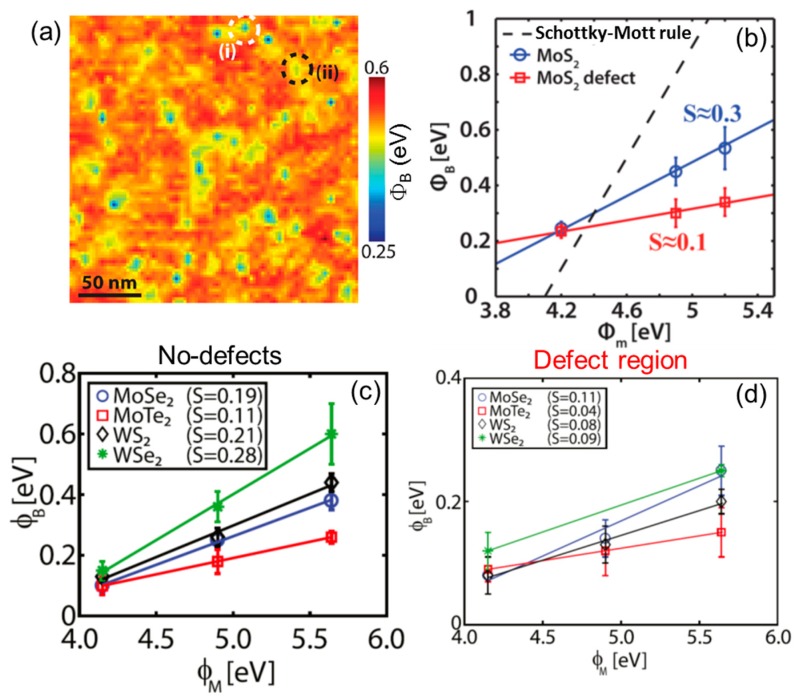
(**a**) Schottky barrier height (SBH) map on the MoS_2_ surface obtained by fitting an array of 128 × 128 local current-voltage (I-V) curves with the thermionic emission model. (**b**) SBH for the defect-free MoS_2_ areas (blue) and on subsurface defects (red) vs. the metal tip work function (Φ_Μ_). The pinning factor S was evaluated as the slope of the linear fit. The dotted line is the standard Schottky–Mott rule. The SBH of different TMDs (MoSe_2_, MoTe_2_, WS_2_, WSe_2_) for defect-free (**c**) and defect regions (**d**) vs. the metal tip work function. Panels (**a**) and (**b**) adapted with permission from Reference [45], copyright from the Chemical Society 2017. Panels (**c**) and (**b**) adapted with permission from Reference [75], copyright from the American Chemical Society 2019.

Figure 5a shows a map of the SBH distribution extracted from an array of 128 × 128 I-V curves collected by the conductive diamond tip on the freshly cleaved MoS_2_ surface. More specifically, the local Φ*_B_* values were obtained by fitting the low forward bias region of the individual I-V curves with the thermionic emission model, as already discussed in Figure 2c. This high resolution SBH map showed minimum Φ*_B_* values of ~0.3 eV corresponding to the subsurface defect regions, whereas a nearly constant SBH of ~0.53 eV was observed in the surrounding areas. As a matter of fact, these SBH values are much smaller than the ideal one (~1 eV) predicted by the Schottky−Mott Equation (2) in the specific case of a boron doped diamond tip (work function Φ_M_ = 5.1 eV) in contact with MoS_2_. Hence, even in the regions without the subsurface defects, a Fermi level pinning occurs, although less intense than on defects. The authors excluded that this Fermi level pinning can be due to the sulfur vacancies, and ascribed this effect to gap states induced by the interaction of the metal tip with MoS_2_, as indicated also by ab-initio calculations [59]. Clearly, the high density of low Φ*_B_* ≈ 0.3 eV patches (separated by a distance of ~10 nm from each other) dominates over the background with higher Φ*_B_* ≈ 0.53 eV and determines the effective SBH. Based on these considerations, the results of this high resolution C-AFM investigation are in agreement with those of the C-AFM study in [44], as well as with SBH measurements on macroscopic contacts [58].

To get a more complete description of the Fermi level pinning effect, the dependence of the local SBH on the metal tip work function was also investigated [45]. Figure 5b shows a plot of the experimental Φ*_B_* values as a function of the metal tip work function both on the defect-free MoS_2_ areas and on the subsurface defect regions. In addition to the conductive diamond tip (Φ_M_ = 5.1 eV), a PtSi tip (Φ_M_ ≈ 4.9 eV) and a highly n-type doped Si tip (Φ_M_ ≈ 4.1–4.2 eV) were used in this experiment. The pinning factor S = dΦ*_B_*/dΦ_M_ was evaluated as the slope of the linear fit of these data. The obtained values for S are ~0.3 and ~0.1 for the MoS_2_ regions without defects and for the regions with subsurface defects, respectively. The observed pinning factor of the defect-free MoS_2_ areas was found to be consistent with the theoretically predicted values for pristine MoS_2_ considering gap states induced exclusively by the metal/TMD interaction [60,61]. On the other hand, the measured pinning factor on the defect sites matches well with the experimentally obtained values on metal/MoS_2_ contacts [58,60,76], confirming the dominant role of these defects on the overall contact behavior.

This kind of investigation was also extended to other TMDs, i.e., MoSe_2_, MoTe_2_, WS_2_, and WSe_2_ [75]. Figure 5c,d shows the SBH values for the different TMDs evaluated in the defect-free (c) and in the defect regions (d) as a function of the metal tip work function. A similar behavior in the case of MoS_2_ is observed also for these TMDs, i.e., the presence of Fermi level pinning in the defect-free regions, ascribed to gap states induced by the metal tip, and an even stronger pinning (30%–40% increase) on defect regions due to defects induced gap states.

### 2.2. Nanoscale Mapping of MoS_2_ Schottky Barrier Tuned by Oxygen Plasma Treatments

As previously discussed, the Fermi level pinning at the metal/TMDs interface has important implications in electronic device performances, since the resulting Schottky barrier ultimately rules the device on-resistance, as well as the type of carriers (n-type or p-type) that can be injected in the channel of TMD-based transistors. In the specific case of MoS_2_, which is an unintentionally n-type doped semiconductor, the Fermi level pinning close to the conduction band results in a relatively small SBH for electron injection and in a high SBH for holes injection. Hence, MoS_2_ transistors commonly exhibit an n-type behavior associated with the formation of an electron accumulation channel for positive gate bias above the threshold voltage, whereas the observation of a p-type behavior related to the hole inversion channel at a sufficiently high negative bias is typically precluded. Clearly, this represents an obstacle for the realization of a complementary MOS technology all based on MoS_2_.

**Figure 6:**
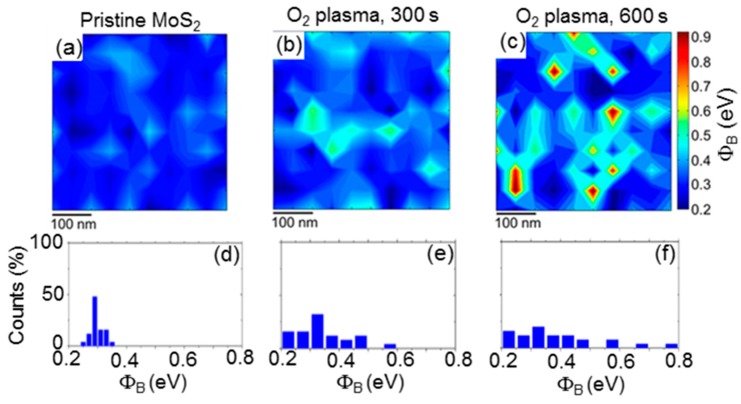
Schottky barrier height maps and histograms on pristine MoS_2_ (**a**,**d**) and after O_2_ plasma for 300 (**b**,**e**) and 600 s (**c**,**f**). Images adapted with permission from Reference [29], copyright from the American Chemical Society 2017.

Recently, soft O_2_ plasma functionalization revealed an effective method to finely tailor the SBH of multilayer MoS_2_, and this approach was exploited to demonstrate MoS_2_ field effect transistors with ambipolar (i.e., both n-and p-type) behavior [29]. In this context, nanoscale electrical analyses by C-AFM showed how the SBH of MoS_2_ can be tuned by increasing the plasma exposure time [29]. Figure 6a,d reports the SBH map and the histogram of the Φ*_B_* values measured on pristine MoS_2_, showing a narrow SBH distribution. Figure 6b reports the SBH map after the 300 s soft plasma treatment, which results in a broader SBH distribution, with Φ_B_ ranging from 0.21 to 0.58 eV, as shown by the histogram of the Φ_B_ values (see Figure 6e). Finally, Figure 6c shows the SBH map after the 600 s soft plasma treatment. In this case, the SBH distribution extends from ~0.2 to ~0.9 eV (see Figure 6f). Hence, it includes both regions with a low barrier for electrons and regions with a low barrier for holes, being the Schottky barrier for holes Φ_B,h_ = E_g_ − Φ_B_, with E_g_ as the bandgap of multilayer MoS_2_.

**Figure 7:**
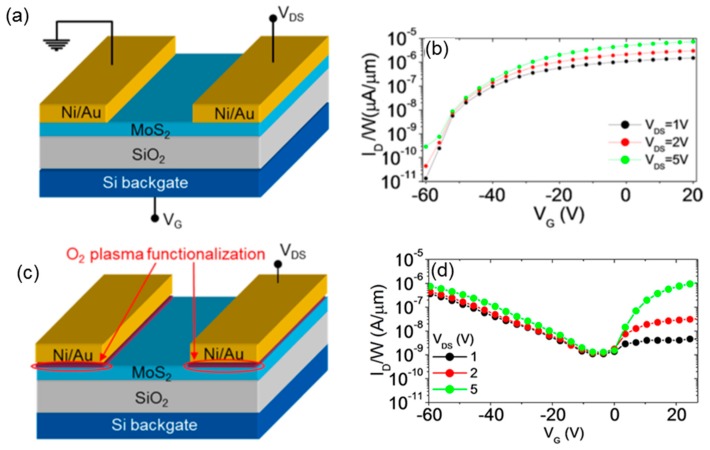
Schematic illustrations of back-gated field-effect transistors with source and drain contacts deposited on pristine MoS_2_ (**a**) or on areas selectively exposed to the O_2_ plasma for 600 s (**c**). I_D_-V_G_ characteristics for a pristine MoS_2_ transistor (**b**) and for a transistor with O_2_ functionalized contact areas (**d**). Adapted with permission from Reference [29], copyright from the American Chemical Society 2017.

Hence, starting from a narrow distribution of low SBHs for electrons in the case of pristine MoS_2_, the SBH map was modified after a 600 s O_2_ plasma treatment into a broad distribution formed by nanometric patches with low SBH for holes in a background with low SBH for electrons. These SBH inhomogeneities in the O_2_ plasma treated samples were associated to lateral variations of the incorporated oxygen concentration in the MoS_2_ surface region [29]. Back-gated FETs were fabricated with Ni source and drain contacts deposited on pristine MoS_2_ (see schematic in Figure 7a) or on areas selectively exposed to O_2_ plasma functionalization for 600 s (see schematic in Figure 7c). Figure 7b shows the transfer characteristics I_D_-V_G_ for different drain bias values (V_DS_ = 1, 2, and 5 V) measured on a FET with channel length of L = 10 μm fabricated on a pristine MoS_2_ flake. The n-type transistor behavior typically reported for MoS_2_ FETs can be observed, with a monotonic increase of I_D_ over more than five decades in the considered gate bias range. Figure 7d shows the transfer characteristics I_D_-V_G_ (for V_DS_ = 1, 2, and 5 V) of a MoS_2_ transistor with the same channel length and thickness, but with the source and drain contacts deposited on plasma O_2_ functionalized regions. For V_DS_ = 1 and 2 V, the transfer characteristics exhibit a pronounced p-type behavior, with the hole current branch (for V_G_ < 0) significantly higher than the electron current branch (for V_G_ < 0), whereas for V_DS_ = 5 V an ambipolar behavior is observed, with the I_on_/I_off_ current ratio of ~10^3^ for both the electrons and holes branches.

The ambipolar behavior in O_2_ functionalized FETs was explained by the coexistence of regions at low SBH for electrons and regions at low SBH for holes within the same source and drain contact areas, as demonstrated in the SBH map of Figure 6c. In particular, for a positively biased drain contact (V_DS_ > 0), the injection of electrons from the source to the accumulation channel (for V_G_ > 0) occurs through regions with lower SBH for electrons, whereas the injection of holes from the drain to the inversion channel (for V_G_ < 0) is allowed by the regions with low SBH for holes. The possibility to have both the n- and p-type current transport in a single device structure is an important step towards the implementation of a CMOS technology with MoS_2_.

## 3. Local Resistance Mapping at Grain Boundaries in CVD Grown MoS_2_

The C-AFM experiments reviewed in the previous section demonstrated the enhanced current injection due to localized defects on the surface or near-surface region of multilayer TMDs [44,45,75]. These defects are responsible for the Fermi level pinning effect commonly observed at metal Schottky junctions with these semiconducting materials.

In addition to these localized defects, the presence of extended defects, such as grain boundaries (GBs), must be taken into account in the case of CVD grown TMDs. As an example, the CVD deposition of MoS_2_ on a commonly used SiO_2_/Si substrate typically results in the formation of a 2D polycrystalline material, composed by several domains with a triangular shape separated by GBs [77]. The structural and electrical properties of GBs have been the object of many experimental and theoretical investigations in the last years [78,79], due to their impact on the mobility of MoS_2_ field effect transistors and their role in peculiar extrinsic charge transport phenomena in MoS_2_ [80,81]. Recent investigations indicated that inter-domains scattering (due to GBs) can play a major role (as compared to the intra-domain scattering due to localized defects [68,82,83]) in the degradation of the MoS_2_ mobility, especially for certain misorientation angles between MoS_2_ domains [81]. To this purpose, complex transistors structures and modeling for electrical data interpretation have been employed to evaluate the impact of the specific GB configurations on the MoS_2_ channel mobility [81]. In this context, C-AFM has been recently employed as a powerful tool for direct probing of the electrical properties of MoS_2_ domains and GBs. As an example, the C-AFM analyses of a few layers of MoS_2_ grown by CVD on sapphire revealed a reduced current injection in the GBs, located in the topmost and in the buried MoS_2_ layers [84].

**Figure 8:**
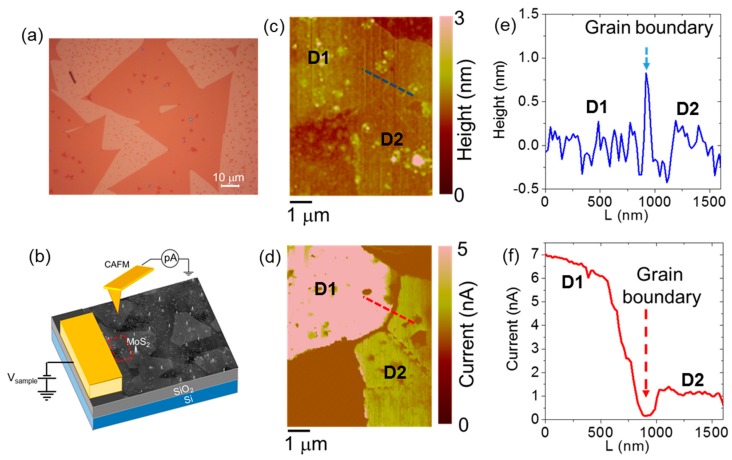
(**a**) Optical microscopy of CVD-grown monolayer MoS_2_ domains on a SiO_2_/Si substrate. (**b**) Illustration of the experimental setup for the local current measurement on MoS_2_ domains by C-AFM. (**c**) Morphology and (**d**) current map measured by C-AFM on a region with two MoS_2_ domains (D1 and D2) separated by a grain boundary. The domain D1 is directly connected to the macroscopic contact. (**e**) Height and (**f**) current line-scans across the grain boundary. Images adapted with permission from Reference [85], copyright from Wiley 2020.

Nanoscale resolution current mapping by C-AFM has been also employed to evaluate the conductance drop associated with GBs in monolayer MoS_2_ grown by CVD onto a SiO_2_/Si substrate [85]. Figure 8a shows an optical microscopy of the as-grown domains, which exhibit typical triangular shapes and sizes up to 50 μm. The coalescence of MoS_2_ domains, resulting in the formation of GBs, can be deduced from this optical image. Figure 8b illustrates the employed experimental configuration for C-AFM measurements, where the current is measured between the nanoscale contact formed by a PtIr tip and a macroscopic electrode partially covering some MoS_2_ domains. Figure 8c,d shows the surface morphology and the corresponding current map measured with an applied bias *V_tip_* = 1 V (referred to the tip) in the region indicated by a red box in Figure 8b. This area includes two MoS_2_ domains (indicated as D1 and D2) separated by a GB, with D1 directly connected to the macroscopic contact. The GB is clearly visible both in the morphology (as a sharp peak with ~1 nm height) and in the current map. The current contrast is almost uniform within the individual domains, whereas a significant conductance drop can be observed from the domain D1 to the domain D2 separated by the GB. This is further elucidated by the two scan lines in Figure 8e,f, extracted from the morphology and current map, respectively. A reduction of the current by about a factor of 7 can be observed moving from D1 to D2, which is clearly associated to the resistance contribution of the GB. Furthermore, a dip in the current can be observed at the GB position. Noteworthy, the width of this current dip is ~150 nm, much larger than the width of the peak in the height profile. This can be explained by a potential barrier associated to the GB, with the formation of depletion regions at the two sides of this barrier. This picture is consistent with the scenario proposed in Reference [81], where a potential barrier ranging from ~0.2 to ~0.6 eV was calculated, depending on the misorientation angle between adjacent MoS_2_ domains.

Hence, C-AFM represents a powerful tool to directly evaluate the impact of GBs in polycrystalline TMDs grown by CVD, without the need for fabricating ad hoc device structures.

## 4. Local Transport Properties in TMD Lateral and Vertical Heterojunctions

### 4.1. Lateral Heterojunctions of TMDs

Due to their unique electronic properties, TMDs offer various solutions to realize lateral, i.e., in-plane, semiconductor heterojunctions. As an example, the significant bandgap variation from monolayer (1L) to bilayer (2L) of TMDs has been exploited as a simple way to create lateral heterostructures at the boundaries between 1L and 2L regions of the same material [86]. Another promising way to tune the TMD electronic structure (i.e., the band gap and the conduction/valence band energies) is the formation of alloys, obtained by mixing Mo with W and S with Se in a controlled way [87,88,89]. As an example, single layers of Mo_x_W_1–x_Se_2_ alloys have shown a tunable band gap, with the conduction/valence band levels depending on the exact ratio of Mo and W [90]. Although Mo and W exhibit a similar electronic structure, preferential segregation of Mo-rich and W-rich regions has been commonly observed during the growth process of the TMD alloys [91], resulting in the formation of one-dimensional (1D) lateral heterojunctions [92].

**Figure 9:**
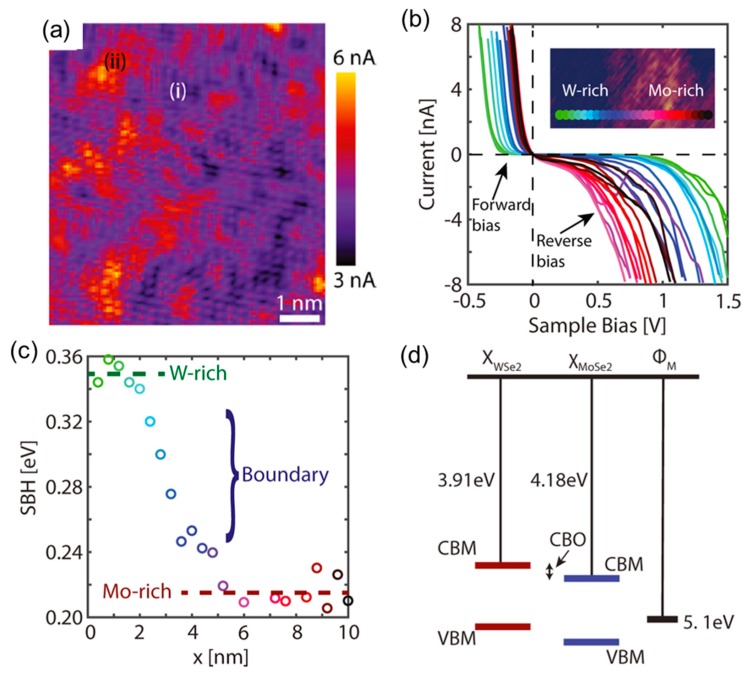
(**a**) C-AFM image of the surface of a Mo_0.3_W_0.7_Se_2_ alloy, showing regions with different conductivities. (**b**) I-V characteristics across a W-rich/Mo-rich boundary (insert); the rainbow line indicates the positions where the I-V characteristics were recorded. (**c**) SBH as a function of the position along the line, with the color code corresponding to the exact I-V curves of panel (**b**). (**d**) Schematic band diagram of a WSe_2_/MoSe_2_ lateral heterojunction, illustrating the conduction band minima (CBM) and valence band maxima (VBM) of WSe_2_ and MoSe_2_ and the conduction band offset (CBO). Images adapted with permission from Reference [69], copyright from the American Chemical Society 2018.

Recently, Bampoulis et al. [69] used C-AFM to characterize the conductivity of a Mo_x_W_1−x_Se_2_ alloy at the nanoscale. They observed the segregation of Mo-rich and W-rich domains and demonstrated that these different regions exhibit distinct SBHs values, reflecting the different band structures of WSe_2_ and MoSe_2_. An atomic resolution C-AFM map of a Mo_x_W_1-x_Se_2_ alloy (with x = 0.3) is reported in Figure 9a, showing distinct differences in the conductivity between neighboring regions with a nanometer size (indicated with (i) and (ii)). To get further insight into this observation, a local I-V spectroscopy was carried out on these differently conductive regions. Figure 9b shows a series of I-V characteristics recorded along the line indicated in the inset which depicts the boundary between the Mo-rich (more conductive) and W-rich regions (less conductive). The I-V curves appear to gradually change when moving from one region to the other. Figure 9c shows the SBH values along this line, extracted by thermionic emission fitting of the individual I-V curves in the forward bias regime. The transition to the smaller SBH (Mo-rich) regions happens gradually and on the length scale of about 3–4 nm. This gradual transition is due to band bending across the phase boundary. The Mo-rich and W-rich regions were expected to approach the electronic band diagram of the MoSe_2_ and WSe_2_ crystals, as schematically illustrated in Figure 9d. Based on this diagram, a type-II staggered gap heterojunction is expected when moving from a Mo-rich region to a W-rich region, and band bending occurs at the interface of the two regions. Finally, assuming that the Fermi level pins at the same energy level across the boundary, a conduction band offset (CBO) of ~0.13 eV was estimated from the SBH difference between the two regions.

### 4.2. Vertical Heterostructures of TMDs

In addition to the 1D lateral heterojunctions discussed in the previous paragraph, 2D semiconducting heterostructures obtained by vertical stacking of different TMDs are currently the object of increasing scientific interest, due to the wide range of potential applications in electronics and optoelectronics [10]. In particular, 2D heterostructures of TMDs with a type II band alignment, such as MoS_2_ and WSe_2_, offer the possibility to implement band-to-band tunneling diodes and transistors for ultra-low-power consumption logic applications.

**Figure 10:**
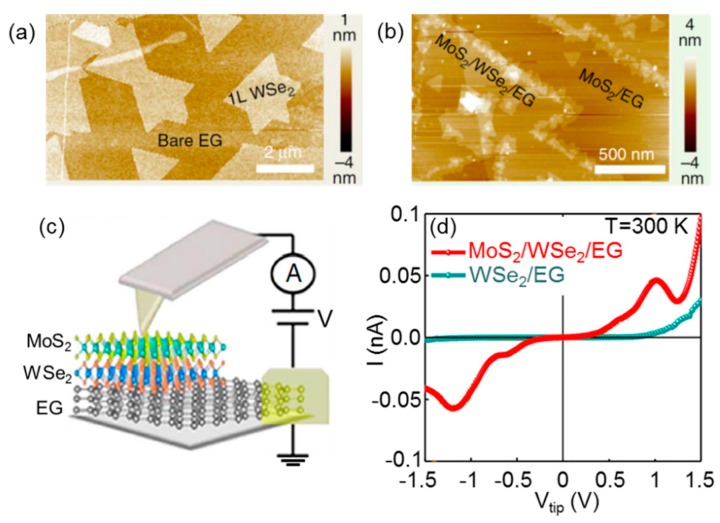
AFM morphology of (**a**) single layer WSe_2_ and (**b**) MoS_2_/WSe_2_ stacks deposited onto epitaxial graphene (EG) on SiC. (**c**) Schematic of the C-AFM setup for the I-V measurement in this layered system. (**d**) I-V curves recorded at room temperature (T = 300 K) on the WSe_2_/EG p-n junction and on the MoS_2_/WSe_2_/EG heterostructure. Images adapted with permission from Reference [12], copyright from the Nature Publishing Group 2015.

Recently, Lin et al. [12] reported the growth of MoS_2_/WSe_2_ heterostructures onto epitaxial graphene (EG) on SiC by sequentially depositing monolayers of the two TMDs employing oxide powder vaporization or metal-organic CVD. In particular, WSe_2_ was first grown on EG at 950 °C. Following this first growth step, the surface coverage of the WSe_2_ on EG was typically >60%, with a lateral size of 2 µm for WSe_2_ domains, as illustrated by the AFM image in Figure 10a. Subsequently, the growth of MoS_2_ on WSe_2_/EG was performed at 750 °C. The MoS_2_ domains are smaller (~300 nm) and typically grow on WSe_2_ starting from the domain’s edges, as illustrated by the AFM image in Figure 10b. Given the small size of the MoS_2_/WSe_2_ areas, C-AFM was employed to investigate a vertical current transport across the heterostructure [12]. In particular, local I-V measurements through the MoS_2_/WSe_2_/EG and WSe_2_/EG heterostructures were carried out at room temperature according to the schematic in Figure 10c. Representative I-V curves collected on the two systems are reported in Figure 10d. The I-V characteristics of the WSe_2_/EG heterojunction exhibit a slight rectifying behavior, associated to the p-n junction between p-type WSe_2_ and n-type EG on SiC. On the other hand, the curves measured on the MoS_2_/WSe_2_/EG show a current peak (at V_peak_ ≈ 1.1 V) followed by a valley (with a peak-to-valley current ratio of 1.9) and, finally, an exponential current increase. The negative-differential-resistance (i.e., the Esaki diode behavior) is a clear evidence of band-to-band-tunneling occurring at room temperature, indicating the formation of an ultra-sharp interface at the MoS_2_/WSe_2_ n-p junction.

### 4.3. Vertical Heterostructures of TMDs with Bulk Semiconductors

As shown in the previous paragraph, the Van der Waals epitaxy of heterostructures entirely made of 2D materials is still in its infancy, and further developments of this approach will be required to realize these systems on a large area. On the other hand, the integration of 2D materials with conventional bulk semiconductors can represent an easier root toward the exploitation of these low dimensional materials in (opto)electronics. In fact, this approach allows adding new functionalities to the existing semiconductor devices, and opening the way to the demonstration of new device concepts [11,32,93]. In the last years, the integration of graphene and semiconducting TMDs with silicon and other semiconductors, such as GaN and related materials [94], has been explored by several research groups. Different approaches have been explored, from the transfer of 2D materials [95,96,97,98] to the direct growth on the semiconductor substrate [19,99]. In particular, the epitaxial growth of MoS_2_ on the basal plane of GaN is especially favored by the low in-plane lattice mismatch (<1%) between the two hexagonal crystals. Furthermore, the small difference in the thermal expansion coefficients between the two materials is expected to result in a reduced strain during cooling down from the growth temperature of MoS_2_ to room temperature [100,101].

**Figure 11:**
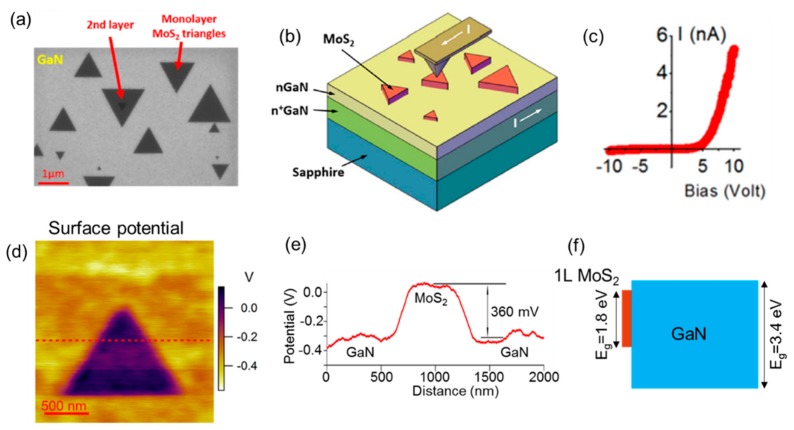
(**a**) SEM image of triangular domains of epitaxial monolayer MoS_2_ grown by CVD on GaN. (**b**) Schematic of the C-AFM setup for local I-V analyses on the MoS_2_/GaN junction. (**c**) Current-voltage characteristic on an individual MoS_2_ domain. (**d**) Surface potential map measured by KPFM on a monolayer (1L) MoS_2_ domain on GaN and (**e**) potential line-scan along the dashed line in the map, showing a 360-mV surface potential difference between 1L MoS_2_ and GaN. (**f**) Energy band alignment at the 1L MoS_2_/GaN interface, as deduced from the surface potential map. Images adapted with permission from Reference [19], copyright from the American Chemical Society 2016.

Recently, Ruzmetov et al. [19] reported the CVD growth of epitaxially oriented MoS_2_ islands on the GaN basal plane. Figure 11a reports a scanning electron microscopy (SEM) image of the as-grown MoS_2_ on the GaN surface, consisting of triangular domains of monolayer MoS_2_ with a typical size of ~1 μm. The sides of these triangles were perfectly aligned with the m-plane (1–100) of the wurzite GaN substrate, indicating the in-plane epitaxial alignment of the GaN and MoS_2_ lattices. Thanks to this rotational order, no evidence of grain boundaries was observed in a larger size of monolayer MoS_2_ islands formed by the coalescence of these small domains. This is a major advantage of MoS_2_ grown on GaN with respect to the more commonly used CVD MoS_2_ on amorphous SiO_2_, which is a polycrystalline material with a large density of grain boundaries. In fact, as discussed in Section 3, grain boundaries are one of the main sources of electron mobility degradation in MoS_2_ [81,85].

C-AFM has been applied in combination with KPFM to investigate the electrical properties of the heterojunction between CVD MoS_2_ and n-GaN, specifically the vertical current flow across the heterointerface and the surface potential [19]. Figure 11b schematically illustrates the C-AFM setup for local current measurements, and Figure 11c reports a current vs. tip bias characteristic on an individual monolayer (1L) MoS_2_ domain, showing a rectifying behavior of the tip/MoS_2_/GaN junction. Figure 11d shows a KPFM surface potential map on a GaN region partially covered by the 1L MoS_2_ domain. A line-scan of the surface potential along the red dashed line in the map is reported in Figure 11e, from which a surface potential difference of ≈360 meV between MoS_2_ and n-GaN can be evaluated [19]. Finally, Figure 11f shows an illustrative energy band diagram showing the type I energy band alignment between 1L MoS_2_ and GaN, as deduced from the surface potential map. This peculiar energy band alignment of the MoS_2_/GaN heterojunction has been recently exploited in various device demonstrators, including Esaki diodes [102] and high responsivity deep-UV photodetectors [103].

## 5. Conclusions and Perspectives

The electronic transport properties of semiconducting TMD layers are strongly dependent on point and extended defects present in their crystalline structure. In this context, the C-AFM technique proved an essential tool to investigate the current transport in these materials at nanoscale. In this paper, recent C-AFM studies on TMDs have been reviewed, discussing the implications of local transport phenomena in the overall behavior of TMD-based devices. The current mapping and I-V spectroscopy by C-AFM allowed clarifying the mechanisms responsible for the Fermi level pinning commonly observed at the metal/TMDs interface. In particular, subsurface defects in the transition metal layer (with a density of 10^10^–10^11^ cm^−2^) have been identified as the main origin of the pinning, although the metal/TMD interaction was also shown to partially contribute to this effect. The impact of extended defects, such as grain boundaries, on the conductivity of monolayer MoS_2_ grown by CVD was also directly imaged by C-AFM. Methods for nanoscale tailoring of the Schottky barrier in MoS_2_, such as oxygen plasma functionalization, have been illustrated, and the changes in the barrier height spatial distribution with the plasma exposure time was imaged at the nanoscale. The application of this approach for the realization of ambipolar MoS_2_ transistors has been also discussed. Finally, C-AFM studies of the current transport in lateral and vertical heterostructures based on TMDs have been reported. As an example, a high-resolution current mapping of Mo_x_W_1−x_Se_2_ alloys showed the formation of 1D lateral heterojunctions between the nanoscale Mo- and W-rich regions formed due to phase segregation. Finally, the C-AFM-based current-voltage spectroscopy provided insight on the current injection mechanisms in vertical heterojunctions formed by van der Waals epitaxy of different TMDs (such as the MoS_2_/WSe_2_ junction) and by the integration of TMDs with bulk semiconductors (such as the MoS_2_/GaN junction). Potential applications of these heterojunctions for novel electronic and optoelectronic devices have been also discussed.

Further progress in the development of 2D/2D or 2D/3D van der Waals heterostructures is expected in the forthcoming years. As a matter of fact, realizing the full potential of these material systems will require understanding and controlling disorder, which can obscure intrinsic properties and hinder device performances [104]. Furthermore, atomic defects or disorder can also be harnessed to provide useful electronic, optical, chemical, and magnetic functions. In this context, the C-AFM technique, in combination with other electrical and optical scanning probe methods, will represent a valuable tool to elucidate the correlation between nanoscale and macroscopic properties of the heterostructures, and will provide a guidance to tailor materials properties.

## Figures and Tables

**Table 1:** Recent progresses in the transition metal dichalcogenide (TMD) materials growth and devices.

Year	Achievements	Ref.
2011	Monolayer MoS_2_ n-type FET with a high-k top gate dielectric	[4]
2011	Integrated logic circuit based on a single-layer MoS_2_	[25]
2012	Monolayer WSe_2_ p-type FET with a high-k top gate dielectric	[26]
2012	Chemical vapor deposition (CVD) of MoS_2_ atomic layers on SiO_2_ substrates	[15]
2014	FET built from all 2D material components	[27]
2014	Vertical and in-plane heterostructures of WS_2_/MoS_2_ monolayers grown by CVD	[13]
2014	Atomic layer deposition (ALD) of MoS_2_ thin films	[22]
2015	CVD of epitaxial monolayer MoS_2_ on sapphire	[18]
2015	Metal organic CVD of a highly uniform monolayer MoS_2_ on 100 mm SiO_2_ wafers	[20]
2015	WSe_2_-based CMOS and integrated circuits	[28]
2015	Tunnel diodes based on MoS_2_/WSe_2_ vdW heterostructures grown by CVD	[12]
2016	MoS_2_ FET with a 1 nm gate length	[7]
2016	MoS_2_ FET with a sub −10 nm channel length	[8]
2016	CVD of epitaxial monolayer MoS_2_ on gallium nitride	[18]
2016	Pulsed laser deposition (PLD) of MoS_2_ thin films	[24]
2017	Ambipolar MoS_2_ FET by tailoring the Schottky barrier with oxygen plasma	[29]
2017	Ohmic contacts to monolayer MoS_2_ by van der Waals bonded metal/h-BN electrodes	[30]
2017	Demonstration of a microprocessor based on CVD grown bilayer MoS_2_	[31]
2017	Hot electron transistor with GaN emitter, graphene base, and WSe_2_ base-collector barrier	[32]
2018	Batch CVD growth of uniform monolayer MoS_2_ on 150 mm soda-lime glass wafers	[21]
2019	Van der Waals contacts between 3D metals and 2D semiconducting TMDs	[33]
